# Molecular mechanisms of heart failure: insights from *Drosophila*

**DOI:** 10.1007/s10741-016-9590-3

**Published:** 2016-12-01

**Authors:** Shasha Zhu, Zhe Han, Yan Luo, Yulin Chen, Qun Zeng, Xiushan Wu, Wuzhou Yuan

**Affiliations:** 1 The Center for Heart Development, Key Lab of MOE for Development Biology and Protein Chemistry, College of Life Sciences, Hunan Normal University, Changsha, Hunan 410081 China; 2 Center for Cancer and Immunology Research, Children’s National Medical Center, 111 Michigan Ave. NW, Washington, DC 20010 USA

**Keywords:** *Drosophila*, Heart failure, Molecular mechanisms, Conserved

## Abstract

Heart failure places an enormous burden on health and economic systems worldwide. It is a complex disease that is profoundly influenced by both genetic and environmental factors. Neither the molecular mechanisms underlying heart failure nor effective prevention strategies are fully understood. Fortunately, relevant aspects of human heart failure can be experimentally studied in tractable model animals, including the fruit fly, *Drosophila*, allowing the in vivo application of powerful and sophisticated molecular genetic and physiological approaches. Heart failure in *Drosophila*, as in humans, can be classified into dilated cardiomyopathies and hypertrophic cardiomyopathies. Critically, many genes and cellular pathways directing heart development and function are evolutionarily conserved from *Drosophila* to humans. Studies of molecular mechanisms linking aging with heart failure have revealed that genes involved in aging-associated energy homeostasis and oxidative stress resistance influence cardiac dysfunction through perturbation of IGF and TOR pathways. Importantly, ion channel proteins, cytoskeletal proteins, and integrins implicated in aging of the mammalian heart have been shown to play significant roles in heart failure. A number of genes previously described having roles in development of the *Drosophila* heart, such as genes involved in Wnt signaling pathways, have recently been shown to play important roles in the adult fly heart. Moreover, the fly model presents opportunities for innovative studies that cannot currently be pursued in the mammalian heart because of technical limitations. In this review, we discuss progress in our understanding of genes, proteins, and molecular mechanisms that affect the *Drosophila* adult heart and heart failure.

## Introduction

Heart failure (HF) is the culmination of diverse cardiac muscle pathophysiological insults resulting in a progressive and deleterious decline in heart function, such that the metabolic demands of the organism are not met. Clinically, this presents as dyspnea, fluid retention, and reduced tissue perfusion with death resulting from lethal arrhythmias or insufficient pump function [1]. The World Health Organization (WHO) has identified cardiovascular disease as the worldwide leading cause of death, and a profound economic healthcare burden. HF is the culmination of cardiovascular disease that can arise from diverse conditions including abnormal heart development or valve formation, coronary atherosclerosis, hypertension, acute pulmonary embolism, or emphysema. External influences, including pregnancy and fatigue, can also cause HF. Because cardiovascular diseases are complex, multifactorial pathologies associated with both genetic and environmental factors [2], the development of new pharmacological and device-based therapies for HF has proven disappointing.

HF is not unique to humans but is observed in many other species, and HF disease models have been developed in rats and mice and in larger mammals including dogs and pigs [3]. The *Drosophila* adult heart which is a linear tube comprising two rows of myocardial cells has recently been used to investigate aspects of cardiac biology relevant to understanding human HF. The *Drosophila* heart can be divided into thoracic and abdominal heart sections [4, 5]. The abdominal heart is divided by internal valves into four chambers which allow hemolymph to enter the heart after a contraction. Hemolymph provides nutrients and hormones to the fly’s internal organs, allowing flies to live for days with a severely damaged heart, because unlike mammals, there is a distinct non-cardiac system (tracheoles) that delivers oxygen to tissues. The myocardium surrounded by non-contractile pericardial cells contains spirally orientated myofibrils. It provides an excellent model with which to dissect out the cell-autonomous and non-autonomous mechanisms of heart failure.

In addition to its comparative simplicity, the *Drosophila* heart displays strikingly conserved structural and functional features which, combined with a much shorter lifespan and an unprecedented wealth of available experimental genetics tools, make it a powerful model system for the insights to explore molecular mechanisms underlying HF. Many genes, proteins, and molecular and cellular pathways involved in cardiac biology are well conserved from flies to humans [6]. These include, for example, highly conserved contractile proteins and ion channel proteins; contractile process-associated proteins. In addition,ion channels including CaMKII, *dSUR*, Ctrl, Ih/HCN [7] and KCNQ [8] are functionally conserved in fly and mammalian hearts. Furthermore, numerous genes regulating cardiac development are functionally conserved from flies to mammals [9], including *Tinman/Nkx2.5*, *Neuromancer/TBX20*, and *Pannier/GATA4*.

Together with the striking molecular and cellular conservation underlying heart development and function, advances in powerful methods allowing high resolution, accurate analysis of *Drosophila* heart biology such as Pacing, OCT (optical coherence tomography) [10], and atomic force microscopy (AFM) [11] also provide a compelling rationale for use of the fly model to elucidate fundamental mechanisms of HF. Insights thus obtained can be used to efficiently direct translational research into increasing costly, time-consuming, and technically challenging vertebrate models en route to clinical interventions.

## Ion channel proteins contribute to heart failure

Ca2+ signaling is a classical pathway in maintenance of adult heart function. Wolf established a genetic method to monitor myocardial Ca2^+^ cycling in *Drosophila*, in which cardiac-specific expression of GCaMP2 acts as a genetically encoded calcium indicator. Ca2^+^ signaling in *Drosophila* myocardium is similar to that of the mammalian heart in several aspects [12]and may reveal promising pathways to address heart disease. Recent investigations show that Ca2+/calmodulin-dependent protein kinase and phosphatase play essential roles in the adult heart. For example, increased free cellular Ca2^+^ activates CaMKII, leading to phosphorylation of proteins involved in Ca2^+^ handling [13]. In *Drosophila*, cardiac-specific inhibition of CaMKII reduces heart rate and increases the incidence of asystole while overexpression of CaMKII increases spontaneous heart rate and reduces arrhythmias [14]. Because CaMKII function is conserved from flies to mammals, the modulation of Ca2^+^ handling via CaMKII targeting may address problems associated with cardiac aging in humans. Additional insights into potential approaches based on conserved pathways come from studies of calcineurin, a calcium/calmodulin-dependent protein phosphatase. Activated calcineurin is necessary and sufficient to drive cardiac hypertrophy [15]. Inhibition of galactokinase causes cardiomyopathy by suppressing activation of calcineurin, and galactokinase has been identified as a novel candidate modifier of calcineurin-induced cardiomyopathy in the fly [16].

Potassium K^+^ channels regulate heart rate and cardiac rhythm in both *Drosophila* and mammals [17]. In *Drosophila*, mutations in the *KCNQ* gene cause cardiac arrhythmias in the adult fly and thus *KCNQ* is protective and important for aging [8]. In addition, the ATP-sensitive K^+^ channel gene *dSUR* protects against heart failure due to stress responses. The expression of *dSUR* is diminished in the aged *Drosophila* heart, and inhibition of *dSUR* in young flies confers an aged heart phenotype. *dSUR* expression is regulated by *Tinman* and the *GATA* transcription factor *Pannier*, both of which are highly conserved cardiac regulatory factors [18].

This reference indicated that dietary copper restriction in rats results in cardiomyopathy and decreases in cytochrome c oxidase as well as decreases in levels of the delta-subunit of ATP synthase [19]. Cu deficiency leads to severe cardiovascular dysfunction including cardiac hypertrophy [20]. Cardiac-specific knockout of *Ctr1*(copper transporter receptor) leads to cardiac hypertrophy in both *Drosophila* and mouse [21].

## Energy homeostasis and heart function

Metabolism of sugars and fats are conserved between mammals and *flies*, and *Drosophila* heart function is affected by high-sugar diet (HSD) and high-fat diet (HFD), as well as time-restricted feeding (TRF) [22]. These results suggest that heart function is closely related to energy homeostasis in *Drosophila*.

Insulin/insulin-like growth factor (IGF) signaling is a well-established genetic pathway regulating longevity [23, 24]. *Drosophila* mutants of insulin-like receptor (*InR*) and *chico* (encoding the insulin receptor substrate) extend the lifespan of the organism as well as protect the heart from decreased resting heart rate and increased heart failure. Additionally, interfering with InR signaling exclusively in the heart, by overexpression of the phosphatase *dPTEN* or the forkhead transcription factor *dFOXO* (negative regulators of insulin/IGF signaling), prevents age-related decline in cardiac fitness. Moreover, the ablation of insulin-producing cells (IPCs) in flies also slows demographic aging and reduces age-dependent heart failure, indicating that both a reduction of insulin receptor signaling and circulating insulin levels influence organismal aging and age-related cardiac susceptibility to pacing stress [24, 25].

Another example showing how alterations in energy homeostasis can be coupled to aging and organ senescence is illustrated by manipulations of the *Drosophila* target of rapamycin (dTOR) pathway. A recent study showed that lowering TOR activity in *Drosophila* prevented age-dependent functional decline of heart performance. The evidence indicates that the Eif4e-binding protein (d4eBP) acts tissue autonomously and downstream of dTOR and dFOXO in the myocardium, where it enhances cardiac stress resistance and maintains normal heart rate and myogenic rhythm. Moreover, d4eBP is sufficient to protect long-term cardiac function against age-related decline and that up-regulation of dEif4e is sufficient to recapitulate the effects of high dTOR or insulin signaling [26].

## EGFR pathway mediated heart failure

RTK (receptor tyrosine kinase) signaling, including EGFR, is essential for maintaining heart function in humans. RTK inhibition provokes dilated cardiomyopathies in mammalian heart models [27]. Recent studies in flies and mammals show that both activation and inhibition of EGFR signaling pathways result in heart failure but involve different mechanisms.

In mammals, ERK regulation of balanced concentric and eccentric cardiac growth is an established model [28]. Concentric hypertrophy, also called diastolic heart failure, is associated with thickening of the heart wall without dilation of the left ventricle. Eccentric hypertrophy, also called systolic heart failure or dilated cardiomyopathy, involves heart chamber enlargement with thinning of walls and poor myocardium contractility. In *Drosophila*, cardiac chamber enlargement is caused by inhibition of rhomboid 3 and the Spitz–EGFR pathway and by inhibition of either the EGF ligand or EGFR [29]. Cardiac-specific activation of EGFR, Ras, or Raf in *Drosophila* causes cardiac hypertrophy with decreased heart chamber lumen and enlarged cardio myocytes, but without changes of cardiomyocyte cell numbers. In *Drosophila,* enlarged cardiac chambers may result from addition of sarcomeres. Enlarged myocytes may be associated with the addition of parallel sarcomeres or increased myofibers [30]. EGF signaling, then, is evolutionarily conserved from flies to mammals and its accuracy is required for maintenance of adult heart function.

## Heart failure associated with stress resistance

Oxidative stress contributes to the pathogenesis of age-related heart failure in the fly, associated with decreased stress resistance [9].

The degeneration driven by oxidation is counterbalanced by several pathways involved in repair of oxidative damage and redox balance. The Nrf2 (nuclear factor E2-related factor 2) pathway is important in this regard. In the mouse, the Nrf2 pathway is associated with repair of damage from inflammatory and autoimmune conditions, neurodegeneration, cancer, and other causes [31]. The Nrf2 pathway is an evolutionarily conserved regulator of longevity from invertebrates to mammals. The activation of Nrf2 signaling extends lifespan in many animal models including *Drosophila* and *Caenorhabditis* *elegans* [32]. However, the molecular mechanisms of its anti-aging function are not clear.

MafS (*Drosophila* small Maf protein), a dimerization partner of Nrf2, is the key component in the Nrf2 stress response. With increasing age, the ability to activate Nrf2 targets for stress resistance progressively declines in *Drosophila.* In aged flies, MafS overexpression protects the heart by preserving the accuracy of Nrf2 signaling [33]. Nrf2 anti-aging function declines in other animals as well, including *Macaca mulatta* [34].

Many studies have addressed the regulatory mechanism involved in oxidative stress. Classically, research into the effects of reactive oxygen species (ROS) focused on cell-autonomous signaling [35]. ROS also act as paracrine signaling mediators of the injury response by diffusing into nearby cells. Paracrine interactions between myocytes and non-myocytes are known to be important for normal myocardium development and function but underlying mechanisms are not well defined [36]. Recent studies suggest that ROS can mediate paracrine interactions in the fly heart under physiological conditions, with ROS generated by pericardial cells regulating myocardial function [37]. Surprisingly, this occurs not through direct intercellular signaling by ROS but indirectly through D-MKK3-D-p38 signaling in pericardial cells by ROS-induced activation, which influences myocardial function via cell–cell communication [36]. Anti-oxidant treatment studies to address aging in mammals have very mixed results. It is therefore not yet clear if this represents a viable treatment strategy to combat heart failure.

## Canonical Wnt signaling and heart failure

The Wnt signaling pathway is an evolutionarily conserved signaling cascade that plays essential roles in embryonic development including heart development [38]. Wnt signaling is also important in adult, stem cell regulation, skeletal muscle regeneration, and cancer progression [39, 40], and recent studies indicated that Wnt signaling may be a novel target for treatment of heart failure. In mice, Wnt/β-catenin signaling contributes to heart failure, characterized by skeletal muscle myopathy, through direct interaction with FOXO. Also, activation of Wnt signaling contributes to fiber type shift toward fatigable fiber in chronic heart failure [41]. In a murine model of myocardial infarction, increased canonical Wnt signaling ameliorates fibrosis and cardiac dysfunction through elevated heme oxygenase-1, adiponectin, and increased angiogenesis [42]. Furthermore, Dickkopf-3 (DKK3), a modulator of Wnt signaling, promotes cardiac protection by interrupting the ASK1-JNK/p38 signaling cascade in mice [43]. Taken together, these results show that novel therapeutic targets for curing heart failure might be found in the Wnt signaling pathway. In *Drosophila*, a body of evidence suggests that Wnt signaling may be less important for adult heart function.

In the fly, *pygo* is essential for maintaining the structure and function of the adult heart but functions independently of Wnt signaling [44, 45]. Cardiac-specific knockdown of *pygo* drastically compromised heart function and structure, but, knockdown of other canonical Wnt signaling components, such as *arm/*β*-Cat* or *pan/TCF*, caused only mild cardiac defects. Also, *pygo* mutants fail to show significant genetic interaction with Wnt signaling components. *Pygo* was also shown to be independent of Wnt signaling in lens development [46] and human cancer [47]. *Pygo* may be associated with histone modification. It was reported that *pygo* could interact with Lgs to form a Pygo-BCL9/Lgs-H3K4me complex to regulate Wnt targets [48, 49], and Pygo also combined with the WDR5 core component of H3K4 histone methyl transferase (HMT) [50], suggesting *pygo* involvement in epigenetic modifications that regulate cardiac function.

## Cytoskeletal remodeling and heart failure

The cortical cytoskeleton in cardiomyocytes which couples sarcomere to the membrane at cell–matrix and cell–cell junctions and translates sarcomeric contraction into cell shortening undergoes remodeling in aging and during heart failure [51]. The sarcomere is the fundamental unit of muscle, consisting mainly of cytoskeletal proteins. In addition, sarcomeric myosin heavy chain (Mhc), troponin T, sarcoglycan, dystrophin, and integrin are critical for normal muscle function. The cytoskeleton is subject to turnover throughout the lifespan in *Drosophila*. Screening for cytoskeletal and associated proteins in *Drosophila* revealed 46 genes needed for muscle function, many not previously reported [52].

Integrins are transmembrane receptors that mediate adhesion between the cell and its external environment (such as the extracellular matrix, ECM). Activation of integrins has an effect on cytoskeletal remodeling [53]. There are also reports that integrin-linked kinase( Ilk) promotes senescence of cardiac cells in the rat. Overexpressing Ilk specifically in cardiac fibroblasts caused cell senescence, while inhibiting Ilk ameliorated senescence-related phenomena [54]. However, other studies came to the opposite conclusion, with Ilk playing a protective role and inhibited Ilk inducing serious cardiac defects sufficient to cause a sudden death [55]. In *Drosophila*, Ilk/integrin was shown to play dual roles in modulating cardiac aging [56], such that overexpression or severe inhibition of Ilk/integrin signaling in young flies caused an accelerated cardiac-aging phenotype, while moderate reduction ameliorated the phenotype. Thus, results from the fly model can confirm the observations from mammalian studies and together show Ilk/integrin signaling important for normal longevity and heart function.

Integrin signaling was reportedly regulated by conserved vertebrate proteins called kindlins [57]. Kindlin-2 was suggested to play a role in the development of cardiac syncytium [58] and this was confirmed in *Drosophila*. There are two orthologues of vertebrate kindlin-2 in *Drosophila*, Fermitin1 and Fermitin2, and silencing both them can cause heart failure due to the inability of cardiomyocytes to form a functional syncytium [59]. Kindlin-2 is structurally and functionally conserved from invertebrates to vertebrates, essential for maintenance of heart function through regulation of integrin signaling.

The integrin-like protein vinculin is reportedly associated with heart failure in humans, and carriers of vinculin missense mutation are more sensitive to HF [60]. In *Drosophila*, cardiac-specific *vinculin* overexpression was associated with increased myocardial shortening velocity, 150% longer median life span, and partial rescue of cardiac deficiency due to cardiac myosin heavy chain knockdown. These observations suggest that vinculin reinforces the myocardial cytoskeleton and positively influences contractility and prolongs life. Kaushik et al. also showed that age-related increase in vinculin is conserved across humans, rhesus monkeys, rats, mice, and *Drosophila* [61].

## Statin mechanisms in the *Drosophila* heart

Statins, such as simvastatin, are a mainstay of cardiovascular disease therapy. Molecular mechanisms are well described, including protein prenylation [62]. In *Drosophila*, simvastatin can protect adult cardiac function, significantly prolong life, reduce arrhythmia, and increase contractility. These functions appear to be associated with down-regulation of protein prenylation, rather than changes in juvenile hormone or ubiquin levels [63]. Moreover, isoprenoid synthesis inhibitors increased *Drosophila* lifespan. In mice, simvastatin down-regulated Ras GTPase prenylation leading to weaker membrane association. Overall, these results provide direct evidence that statins protect cardiac function and prolong life span by reducing protein prenylation.

## CCR4-Not complex and chromatin remodeling

The CCR4-Not complex is evolutionarily highly conserved, with roles in chromatin transcriptional activation, RNA deadenylation, and microRNA-mediated mRNA degradation [64–66]. A frequently occurring Not3 SNP is correlated with abnormal cardiac QT intervals, which cause arrhythmias. In *Drosophila*, recent studies confirm that RNAi-mediated silencing of the CCR4-Not components Not3 and UBC4 in adult flies induced myofibrillar disarray and dilated cardiomyopathy [67]. In mice, *not3* ^*+/−*^ heterozygotes exhibit spontaneous cardiac contractility defects and greater susceptibility to heart failure [68]. A link to epigenetic chromatin remodeling was suggested by reversal of these defects through inhibition of HDACs.

## Scox/Sco and apoptosis in cardiomyopathy

In humans, *Sco1* and *Sco2* gene mutations resulting in cytochrome C oxidase (COX) deficiency are associated with cardiomyopathy [69]. *Drosophila* has a single orthologue of *Sco1* and *Sco2*, called *Scox*. Heart-specific knockdown of *Scox* induced dilated cardiomyopathy and reduced adult fly lifespan. It was shown that p53-dependent apoptosis was directly implicated in development of the fly cardiomyopathy. In *Sco2* knockout mice apoptosis is increased in the muscle and liver, strongly implicating cell death in COX deficiency-associated cardiomyopathy caused by *Sco* gene mutations in humans [70].

## Neurodegenerative disease and heart function

Epidemiological evidence reveals an association between HF and neurodegenerative disease. The mechanisms by which certain genes may underlie this linkage have been studied in *Drosophila*. *Presenilin* gene mutations lead to early-onset familial Alzheimer’s disease and can cause dilated cardiomyopathy. In flies, either knockdown or overexpression of the *Drosophila* orthologue of mammalian *Presenilin* (*dPsn*) increased age-related cardiac arrhythmias and both myofibrillar and mitochondrial degeneration [71]. Altering *dPsn* also affected key calcium signaling genes such as inositol 1, 4, 5-triphosphate receptor (*dIP3*R), *dSERCA*, and *RyR* gene [24].

Huntington’s disease (HD), caused by expanded Huntingtin protein’s polyglutamine (PolyQ) repeats, is associated with both cardiovascular events including heart failure and amyloid-like inclusions, and heart failure causes high mortality among HD patients [72, 73]. Research in the *Drosophila* heart model provides insights into molecular mechanisms of HF induced by amyloid protein. Both ROS stress response pathways and amyloid protein unfolding can mediate the detrimental effects of PolyQ in the *Drosophila* heart [74].

One of the main pathologic processes associated with Parkinson’s disease and cardiomyopathy is functional disorder of PTEN-inducible kinase 1 (PINK1), Parkin, which mediates mitophagic elimination of damaged or senescent mitochondria [75]. In *Drosophila*, knockout of *Parkin* and cardiac-specific *Parkin* suppression both caused cardiomyopathy and mitochondrial abnormalities. This was completely prevented by suppressing cardiomyocyte mitochondrial fusion suggesting a central role of mitochondrial fusion in the cardiomyopathy caused by impaired mitophagy [76].

## Conclusion

Despite clear anatomical differences between the invertebrate and vertebrate hearts, many key processes and regulatory mechanisms driving cardiac development and function are evolutionarily conserved from *Drosophila* to humans. Thus, the fly heart can be used to model HF mechanisms.

Abnormal ion channels contribute to heart failure, and inhibition of CaMKII reduced spontaneous heart rate and increased the incidence of asystole. Copper (Cu) is required in cardiac tissue mitochondrial oxidative phosphorylation to provide energy for cardiac contraction. K^+^ channels are conserved in regulating heart rate and rhythm in both *Drosophila* and mammals. The strong conservation of energy metabolism including IGF and dTOR signaling extends to the regulation of obesity, as well as effects on adult cardiac function. Furthermore, the interactions between IGF and dTOR signaling were discovered in *Drosophila*. Cardiac ROS, increased by a high-calorie diet, also plays an important role in HF. *Drosophila* studies have revealed distinctions in Wnt signaling pathway contributions to adult heart function, suggesting the emergence of epigenetic mechanisms of target gene activation. Statins prolong lifespan and protect adult cardiac function by reducing protein prenylation. The study of heart failure may also contribute to understanding of molecular mechanisms of neurodegenerative diseases.

From the standpoint of advancing therapeutic interventions to treat HF, *Drosophila* is the model platform *par excellence* for the design and conduct of whole animal, in vivo screening approaches to identify small molecules targeting key genes, proteins, and pathways in the development of heart disease.
